# Lithium Recycling from Spent NCM523 Cathode and Resynthesis of High‐Nickel Lithium‐Ion Battery Using Recovered Material

**DOI:** 10.1002/cssc.202501510

**Published:** 2025-10-28

**Authors:** Jinwon Seo, Hyerin Jeon, Hui‐il Nam, Jinhee Lee, Yong‐Wook Choi, Jinsub Choi

**Affiliations:** ^1^ Department of Chemistry and Chemical Engineering Inha University Michuhol‐gu Incheon 22212 Republic of Korea; ^2^ Energy System Group Korea Institute of Industrial Technology (KITECH) Sasang‐gu Busan 46938 Republic of Korea

**Keywords:** high‐energy ball milling, lithium extraction, lithium‐ion batteries recycling, nickel–cobalt–manganese oxide cathodes, spent lithium‐ion battery

## Abstract

Many approaches to recycle lithium nickel**–**cobalt–manganese oxide (NCM) cathodes from degraded lithium‐ion batteries (LIBs) have been reported, but they remain challenging due to environmental issues such as pollution or toxic waste. In this study, a sustainable and green approach for lithium recovery and reuse via mechanochemical method based on high‐energy ball milling is employed to extract lithium from spent NCM without high temperature or toxic solution. The recycling process is as follows: planetary ball milling with spent NCM523 cathodes, lithium extraction as a carbonate through subsequent reaction in deionized water. Then the recovered lithium is used to synthesize a high‐nickel NCM cathode material with a freshly prepared NCM precursor. Structural and chemical analyses revealed that the extracted lithium has 11.06% fluorine impurities in an agglomerated form. Consequently, the resynthesized NCM with recycled lithium (RNCM) has relatively low electrochemical performance compared to NCM with reagent lithium (HNCM), achieved a specific capacity of 80.63 mAh g^−1^ at 0.5 C and retains 36.7% capacity after 300 cycles. These findings suggest the feasibility of green recycling of commercial spent LIBs. Subsequent research to eliminate fluorine impurities may mitigate the environmental limitations associated with the current recycling process.

## Graphical abstract

This study investigates an environmentally sustainable means for recovering valuable resources (Li‐salt) including remanufacturing batteries with recovered materials by recognizing the problems of the existing methods used to recover valuable resources from waste batteries.

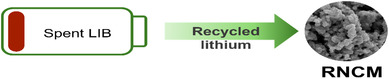

## 1 Introduction

The global demand for lithium‐ion batteries (LIBs) has been rapidly increasing, with the industry expanding ≈30% per year because of the growth of the electric vehicles market. It is more and more important to develop efficient and sustainable recycling technology of wasted batteries, because the disposal amounts of batteries have been steadily increasing as industrial growth.^[^ ^1^, ^2^, ^3^, ^4^ ^]^ Especially, only 10% of discarded LIBs are recycled, while the remaining 90% are either landfilled or stockpiled. Such disposal methods bring significant environmental risks as the heavy metals, electrolytes, and binders within LIBs can severely contaminate soil and water. Consequently, there is a growing need to recover valuable resources from spent LIBs.^[^ ^1^, ^5^, ^6^ ^]^ Among LIB components, LiNixCoyMn_1‐x‐y_O_2_ (NCM) cathode materials are of particular interest due to their high content of valuable metals such as nickel (Ni), cobalt (Co), manganese (Mn), and lithium (Li).^[^ ^7^, ^8^, ^9^ ^]^

Conventional recycling approaches for spent LIBs are categorized as pyrometallurgical and hydrometallurgical methods. Pyrometallurgical process includes high‐temperature treatments, typically ranging from 600 to 1400 °C for recovering valuable metals. In addition, this method uses roasting/calcination techniques with additives (e.g., carbothermic reduction or sulfation) or smelting techniques that makes recovered metals molten via reduction. While the pyrometallurgical process is relatively simple in terms of operational steps, it requires a large amount of energy consumption and capital investment due to the presence of melting process. Moreover, it does not focus on Li but transition metal during metal recovery process.^[^ ^10^, ^11^, ^12^ ^]^ Hydrometallurgical process is that cathode materials could be dissolved in acid or alkaline solution and subsequently recovered as metal salt through solvent extraction and precipitation. This method has benefits such as lower energy consumption and higher metal recovery efficiencies than pyrometallurgical method. However, it involves the use of hazardous chemicals and brings about secondary waste streams, resulting in both economic and environmental concerns.^[^ ^13^, ^14^, ^15^ ^]^

Recently, various approaches have been explored for the recycling of cathode materials. Some studies have reported the extraction of lithium from spent cathodes via sulfurization, followed by resynthesis of NCM. Other methods include dissolving mixed cathode materials such as spent NCM/LCO/LMO in a solution of H_2_O_2_ and H_2_SO_4_ to synthesize new precursors, or applying carbothermal reduction to spent NCM523/LFP, followed by water leaching to recover lithium. These processes aim at lithium extraction, metal recovery, and cathode resynthesis. However, many of these methods still require high temperatures (up to ≈950 °C) or involve the use of strong acids, which pose environmental concerns.^[^ ^16^, ^17^, ^18^ ^]^

To address the obstacles of the above‐mentioned processes, Dolotko et al. proposed a novel mechanochemical method using high‐energy ball milling by facilitating lithium extraction from cathode materials through reductive reactions without the use of high temperatures or chemical agents.^[^ ^19^ ^]^ While the mechanochemical recovery demonstrated a promising means for eco‐friendly recycling, there are limitations that reagent‐grade cathode materials and fresh aluminum (Al) foils should be used for the proposed method.

Herein, we intensively explored a sustainable and scalable method to recycle commercial NCM523 cathode materials by applying a mechanochemical reaction with the planetary ball milling, motivated from an already reported study.^[^ ^19^ ^]^ The planetary ball‐mill process led to the release of Li during the process by reducing the transition metal materials placed in NCM with the use of Al current collector playing as a reductant. The released Li was subsequently obtained as a salt type through the extraction between grinded powder and deionized (DI)‐water with heating (≤95 °C). The finally recovered lithium salt was identified as carbonate form (suitable for cathode synthesis). It was subsequently employed in the synthesizing of new NCM811 materials via calcination with a hydrothermally synthesized precursor as resynthesized NCM (RNCM). It was revealed that RNCM exhibited lower electrochemical performance compared to the reference NCM (denoted as HNCM) due to impurities in the recovered lithium carbonate. Nevertheless, our novel method presents a practical and green strategy for recycling commercial spent cathode materials. This also suggests that valuable resources in spent application should be recovered without the use of toxic chemicals or high energy (introducing high temperature) for doing remanufacturing electrochemical application.

## 2 Results and Discussion

### 2.1 Recycling Lithium via High‐Energy Ball Milling from Spent LIB

**Figure 1:**
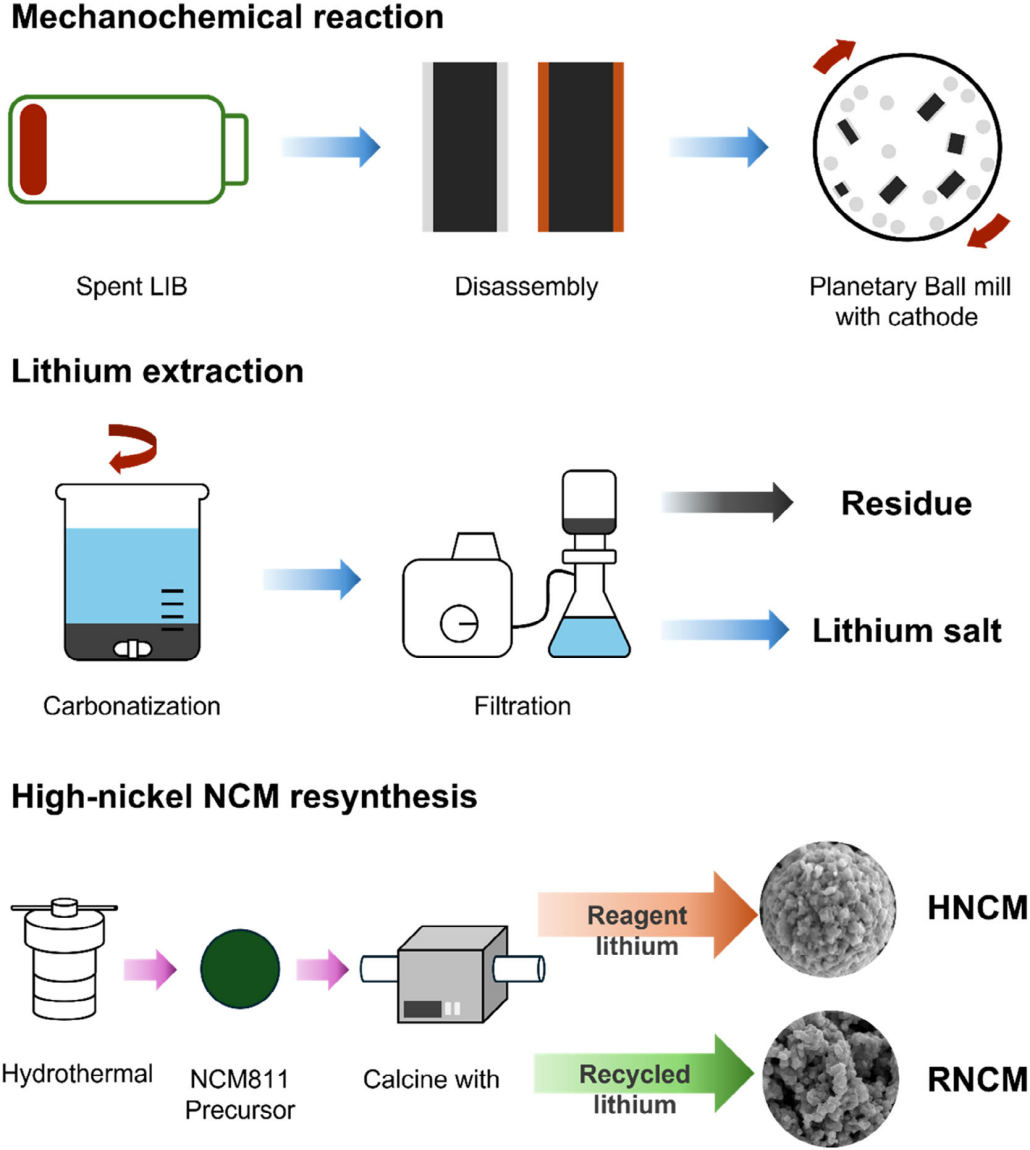
Scheme of LIB recycling and NCM resynthesizing process.

In order to recover lithium from spent LIB as eco‐friendly method, it should be consisted of different processes compared to conventional means such as pyrometallurgical and hydrometallurgical methods. Above all, the use of toxic chemicals and the requirement of high temperature heating are certainly avoided as much as possible. **Figure** **1** summarizes the overall process, carried out in this work, for recovering lithium from spent LIB via sustainable means including resynthesis of NCM battery with recovered lithium salts. Cathode materials were separated from spent 18 650 LIB through charge/discharge cycles and immersion in NaCl solution with drying step (details in Supporting Information). In order to obtain lithium salt such as Li_2_CO_3_ from separated cathode materials, the presented metal recovery process starts with the high‐energy ball milling stage. This ball milling is well known as mechanochemial method because it can make input materials (solid state powders/particles) lead to chemical transition (herein reduction reaction) without any solvents. In addition, the subsequent step (carbonization) could lastly convert to Li_2_CO_3_ from intermediates such as LiAlO_2_ with CO_2_ in ambient air and greatly lower heating temperature (below 100 °C) than conventional recovery method (over 600 °C).

As the first step in metal recovery process, fully discharged cathode should be put in high‐energy ball milling. According to a previous study, the reaction between aluminum current collector and cathode materials during ball milling are followed as^[^ ^19^ ^]^

(1) $${\text{2LiTMO}}_{2}+\text{2Al}\to {\text{TM}+\text{Li}}_{2}{O+\text{Al}}_{2}O_{3}\;\left(\text{TM: Transition metals}\right)$$

(2) $${\text{Li}}_{2}{O+\text{Al}}_{2}O_{3}\to {\text{2LiAlO}}_{2}$$

Additionally, the following reactions occur during the carbonatization step

(3) $${\text{4LiAlO}}_{2}+{\text{9H}}_{2}{O+\text{2CO}}_{2}\to {\text{Li}}_{2}{\text{Al}}_{4}\left({\text{CO}}_{3}\right){\left(\text{OH}\right)}_{12}\cdot {\text{3H}}_{2}{O+\text{Li}}_{2}{\text{CO}}_{3}$$

(4) $${\text{Li}}_{2}{\text{Al}}_{4}\left({\text{CO}}_{3}\right){\left(\text{OH}\right)}_{12}\cdot {\text{3H}}_{2}O\to {\text{Li}}_{2}{\text{CO}}_{3}+{\text{2Al}}_{2}O_{3}+{\text{9H}}_{2}O↑$$

These reactions indicate that aluminum reduces the transition metals included in the cathode during ball milling, leading to the release of lithium. The extracted lithium was subsequently converted to lithium carbonate (Li_2_CO_3_) during carbonatization because of the presence of water and carbon dioxide. A byproduct, lithium aluminum carbonate hydroxide hydrate (Li_2_Al_4_(CO_3_)(OH)_12_·3H_2_O, denoted as LACHH), might also be formed in this step and be able to decompose into Li_2_CO_3_ upon heating. Thus, the ball milling step converts lithium into a chemically reactive form, which can subsequently be recovered as lithium carbonate via aqueous reaction.

The overall recycling process consists of two main steps: ball milling and carbonatization. We initially focused on optimizing the parameters of the ball milling step. Since the milling power is proportional to the number of balls when the sample mass remains constant,^[^ ^20^ ^]^ it is required to investigate sufficient energy input for the mechanochemical reaction by tuning the ball‐to‐powder ratio (BPR) varied from 1:20, 1:30, and 1:40. Figure 1 exhibited the correlation among different BPR values corresponding to recycled samples.

**Figure 2:**
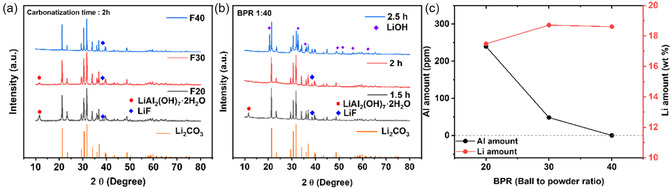
XRD patterns of powders collected after recycling process a) at different BPR and b) at different carbonatization times. c) Quantitative analysis of Al and Li determined by ICP‐OES and AAS.

In **Figure** **2**a, X‐ray diffraction (XRD) results after carbonatization with varying BPR are shown. The presence of Li_2_CO_3_ (JCPDS 87‐0728) was detected, confirming the effectiveness of the proposed process even when applied to spent commercial NCM materials. Peaks corresponding to LiAl_2_(OH)_7_·H_2_O (JCPDS 40‐0710) were detected in the F20 and F30 samples, but not in F40, indicating that insufficient milling power at lower BPRs led to incomplete reactions and impurity formation. The impurity phase LiAl_2_(OH)_7_·H_2_O could be converted to LiAlO_2_ at around 600 °C.^[^ ^21^ ^]^ Due to the extremely high melting point of LiAlO_2_ (≈1784 °C), it may act as an undesirable contaminant during the subsequent synthesis of high‐nickel NCM cathode materials.^[^ ^22^ ^]^ In addition, a peak near 38° corresponding to LiF (JCPDS 04‐0857) was observed. Furthermore, to study the effect of carbonatization time in Li_2_CO_3_ formation, a recycling process was conducted with varying carbonatization times for 1.5, 2, and 2.5 h in Figure 2b. When the carbonatization time was deficient (1.5 h), impurity peaks related to Al‐containing phases, which are similar to those obtained in the F20 and F30 samples (Figure 2a), were still visible. Conversely, when reaction time is excessive (2.5 h), the mixed phase, which contains LiOH (JCPDS 32‐0564) and Li_2_CO_3_ are formed. Due to the difference of reaction mechanisms and onset temperatures of both LiOH and Li_2_CO_3_ during NCM synthesis, it is important to get solo chemical with high purity because mixed precursors should make lithium incorporation significantly different in uniformity and timing. This mismatch may lead to inhomogeneous lithium distribution and cation disorder, potentially compromising the structural integrity and electrochemical performance of the resulting material.^[^ ^23^ ^]^ Meanwhile, quantitative analyses (Figure 2c) confirmed that aluminum content decreased with increasing BPR, and no aluminum was detected in the F40 sample. In addition, atomic absorption spectroopy (AAS) analysis of Li revealed that the lithium content remained relatively constant regardless of BPR. Based on these findings, the optimal BPR and carbonatization time is determined to be 1:40 and 2 h. However, F40 still contains ≈11 mol% of fluorine according to IC analysis (**Table** **1**).

**Table 1:** Ion chromatography (IC) results of F40.

	F^−^ [mg kg^−1^]	F^−^ [wt%]	F^−^ [mol%]
F40	31 162	3.1162	11.06

**Figure 3:**
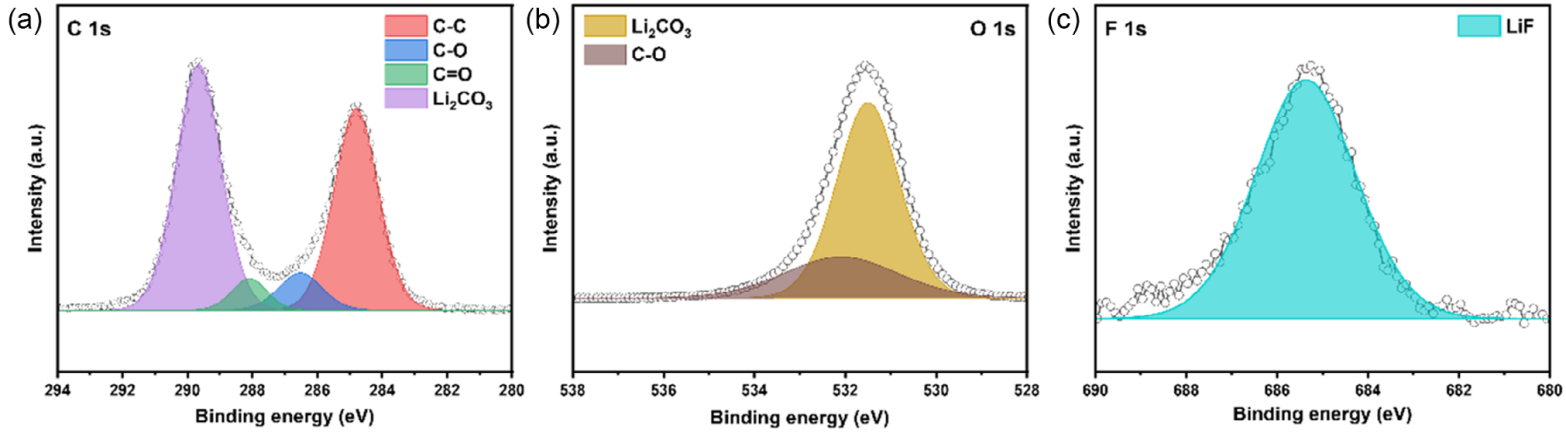
a) C 1s, b) O 1s, and c) F 1s XPS spectra of F40.

Chemical states of F40 were confirmed by X‐ray photoelectron spectroscopy (XPS) analysis. The XPS spectra were calibrated using the C—C bond at 284.4 eV in the C 1s spectrum. As presented in **Figure** **3**a–c, peaks observed around 289.7 eV in the C 1s region and 531.6 eV in the O 1s region both indicated Li_2_CO_3_. Meanwhile, the F 1s spectrum exhibited a peak near 684.8 eV, which was consistent with LiF, supporting the XRD results that suggested the presence of fluorinated impurities.^[^ ^24^, ^25^, ^26^ ^]^ In addition, it could be concluded that the F40 case is the optimal process because there is no significant difference among F20, F30, and F40 powders by confirming both Li_2_CO_3_ and LiF peaks (as shown in Figure S1, Supporting Information).

**Figure 4:**
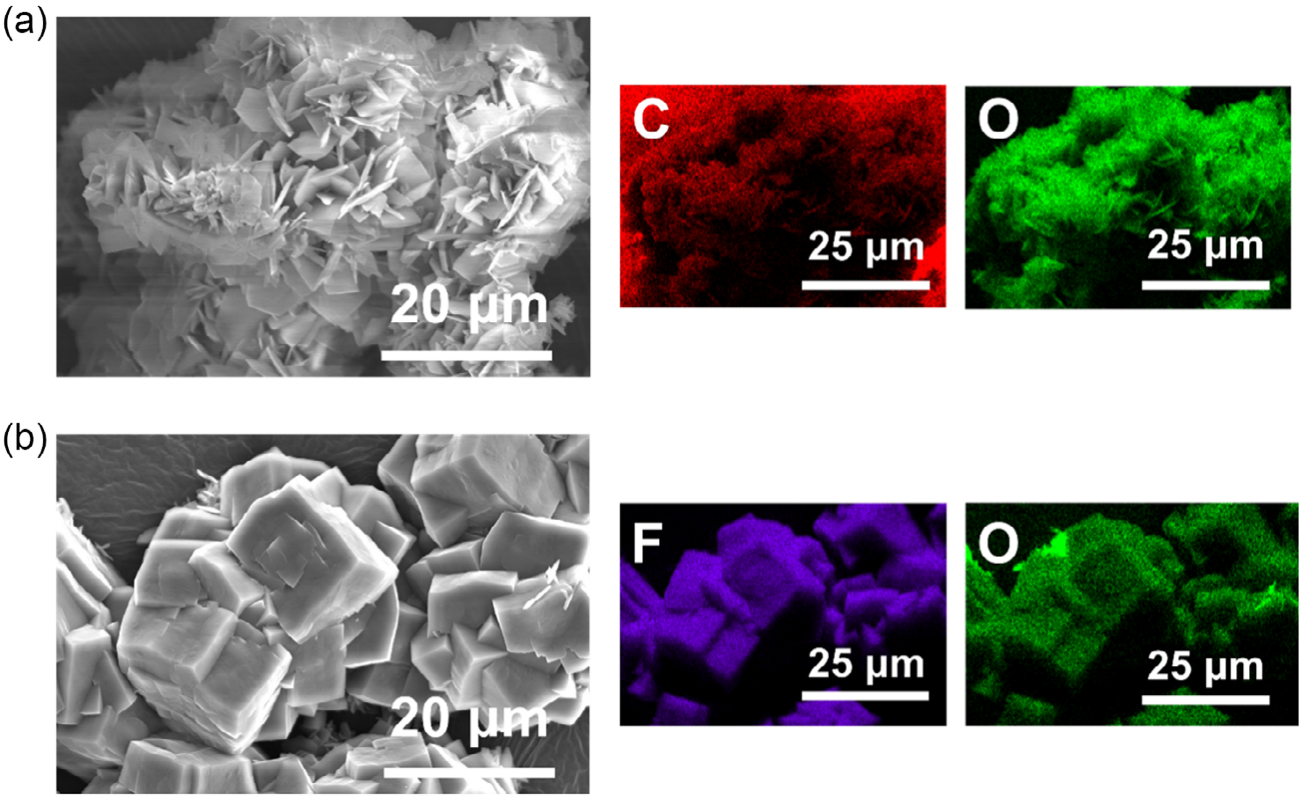
a,b) SEM images and EDS mapping of F40 at different sites.

As depicted in **Figure** **4**, the morphology of F40 powder was investigated. Although all images were taken from the same sample, distinct morphological differences were observed across different sites. According to the corresponding EDS maps, these variations are attributed to heterogeneity in chemical composition, particularly between F‐rich and C‐rich areas. To further substantiate this observation, Figure S2, Supporting Information showed that both F‐rich and C‐rich domains were simultaneously observed within the same regions. Quantitative EDS analyses of each domain confirm significant compositional differences, thereby validating the coexistence of heterogeneous regions within the recycled Li_2_CO_3_ powder. The combined results of this observation, XRD and XPS analyses suggest the coexistence of LiF and Li_2_CO_3_ in the extracted powder.

### 2.2 Synthesis of High‐Nickel Cathode Using Recycled Li_2_CO_3_ and its Properties

Since Li_2_CO_3_ is a commonly used lithium source for synthesizing NCM cathode materials,^[^ ^27^, ^28^, ^29^, ^30^, ^31^ ^]^ we explored the feasibility of using F40 as a lithium source for high‐nickel NCM synthesis as an application. Two high‐nickel NCM (LiNi_0.8_Co_0.1_Mn_0.1_O_2_) were prepared using the same precursor but with different lithium sources (Figure 1): reagent‐grade Li_2_CO_3_ (HNCM) and F40 (RNCM).

**Figure 5:**
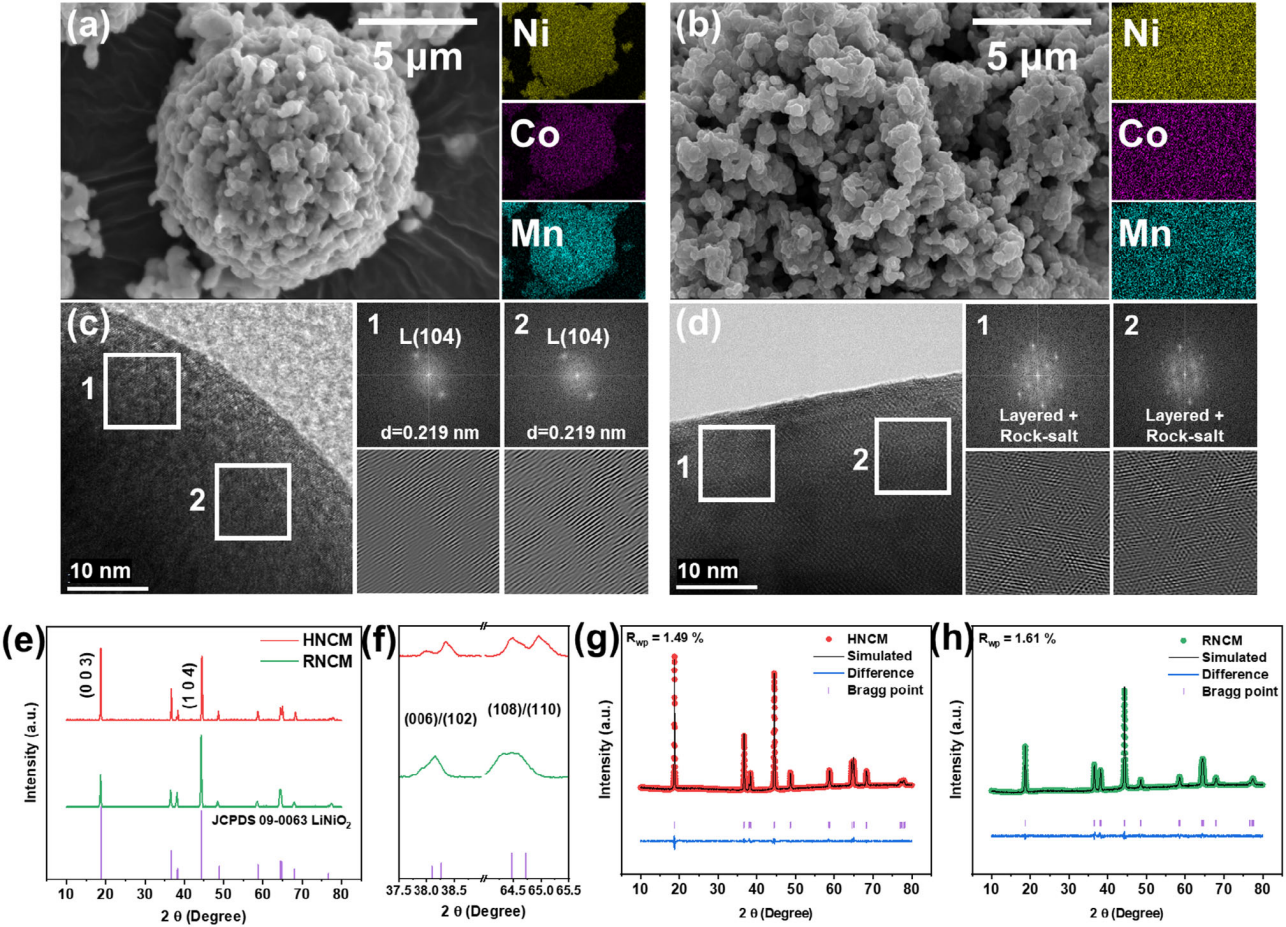
SEM images and EDS mapping of a) HNCM and b) RNCM. FE‐TEM with FFT patterns of c) HNCM and d) RNCM. e,f) XRD patterns and magnified view of (006)/(102) and (108)/(110) peak region of HNCM and RNCM. Rietveld refinement results of g) HNCM and h) RNCM.

Scanning electron microscopy (SEM) analysis showed notable differences in particle morphology between HNCM and RNCM. As shown in **Figure** **5**a, HNCM exhibited well‐defined spherical secondary particles, while RNCM (Figure 5b) mostly consisted of agglomerated primary particles, without the formation of distinct secondary particles. According to energy dispersive X‐ray spectroscopy (EDS) results, the transition metal compositions were similar; HNCM contained Ni 81.64%, Co 10.37%, and Mn 7.98%, whereas RNCM contained Ni 80.03%, Co 10.59%, and Mn 9.38%. In Figure 5c and d, we also compared the crystal structures between HNCM and RNCM. In layered NCM structures, the splitting of (006)/(102) peaks near 38° and (108)/(110) peaks near 64.5° is commonly used as an indicator of well‐developed layered ordering.^[^ ^32^ ^]^ For HNCM, clear splitting was observed in both regions, exhibiting a great layered structure (Figure 5d). In contrast, RNCM exhibits merged (006)/(102) and (108)/(110) peaks, indicating severe cation mixing that hinders the formation of a well‐defined layered structure. This suggests that the alternating arrangement of Li and transition metal (TM) layers is disrupted, which in turn negatively affects the electrochemical performance.^[^ ^33^ ^]^

These morphological differences are originated from the lithium based chemical structure, such as recycled Li_2_CO_3_ (F40) containing 11.06 mol% fluorine, as shown in Table 1. The presence of F species could lead to the environment about Li‐deficient synthesis (≈90 mol% Li) with the decrease of available Li amount. These Li‐deficient conditions induced unfavorable conditions for handling materials for Li‐battery: 1) overconsumption of recycled Li due to the incorporation of Li into the precursor during calcination, 2) nonoccurrence of liquid‐phase sintering, and 3) suppression of the secondary particle both coalescence and growth from small primary particles included in the RS‐layered composite.^[^ ^34^ ^]^ In contrast, unreacted lithium salt persists as a molten phase around primary particles, resulting in liquid‐phase sintering and thereby promoting secondary‐particle formation in the case of Li‐excess conditions (Li/TM > 1.0).^[^ ^34^ ^]^

FE‐TEM (field‐emission transmission electron microscopy; as shown in Figure 5c,d) further corroborates this mechanism. HNCM displays well‐ordered layered lattice fringes with fast Fourier transform (FFT) patterns indexable to the R3m layered structure, indicative of good Li/TM ordering in the bulk. By contrast, RNCM exhibits the coexistence of layered and rock‐salt domains within the particle interior, as evidenced by simultaneous R3m and Fm3m reflections in the FFTs, revealing bulk level Li/Ni intermixing (cation mixing) that disrupts layered ordering and impedes secondary‐particle growth. Such layered–rock‐salt intergrowth under Li‐deficient calcination is consistent with prior observations that Li deficiency yields small primary particles composed of intergrown layered/rock‐salt phases, whereas Li excess promotes liquid‐phase sintering and secondary‐particle formation.^[^ ^34^ ^]^

In Figure 5e and f, we also compared the crystal structures between HNCM and RNCM. In layered NCM structures, the splitting of (006)/(102) peaks near 38° and (108)/(110) peaks near 64.5° is commonly used as an indicator of well‐developed layered ordering.^[^ ^32^ ^]^ For HNCM, clear splitting was observed in both regions, exhibiting a great layered structure. In contrast, RNCM exhibits merged (006)/(102) and (108)/(110) peaks, indicating severe cation mixing that hinders the formation of a well‐defined layered structure. This suggests that the alternating arrangement of Li and TM layers is disrupted, which in turn negatively affects the electrochemical performance.^[^ ^33^ ^]^

Another key factor in high‐nickel NCM performance is the degree of cation mixing. NCM belongs to the α‐NaFeO_2_‐type layered structure, characterized by alternating TM and Li layers. However, Ni^2+^ (0.69 Å) has a similar size to Li^+^ (0.72 Å) and a relatively low migration energy barrier, making it prone to occupying Li layer.^[^ ^35^ ^]^ We can evaluate the degree of cation mixing by using the intensity ratio of the (003) and (104) peaks intensity (I_003_/I_104_) in XRD patterns. This ratio indirectly reflects the degree of Li^+^ and Ni^2+^ ordering within the lattice.^[^ ^36^ ^]^ The I_003_/I_104_ ratio for HNCM was ≈1.12, while RNCM has a significantly lower value of 0.44, suggesting severe cation mixing (**Table** **2**). This property observed in RNCM is likely attributed to excessive fluorine doping originating from F40. The fluorine molar fraction of F40 is calculated to be 11.06% (Table 1), which is equivalent to an estimated the F doping level of ≈4 mol% in the synthesized RNCM. Qiu et al.^[^ ^37^ ^]^ reported that when F doping level in NCM exceeds 2 mol%, O^2−^ is excessively substituted by F^−^, resulting in an increased reduction of Ni^3+^ to Ni^2+^ in order to maintain charge neutrality. The increased Ni^2+^ content leads to increased cation mixing.

**Table 2:** Rietveld refinement results of HNCM and RNCM.

	HNCM	RNCM
a [Å]	2.867	2.882
c [Å]	14.20	14.21
V [Å^3^]	101.29	102.22
c/a	4.95	4.93
I_(003)_/I_(104)_	1.12	0.44

**Figure 6:**
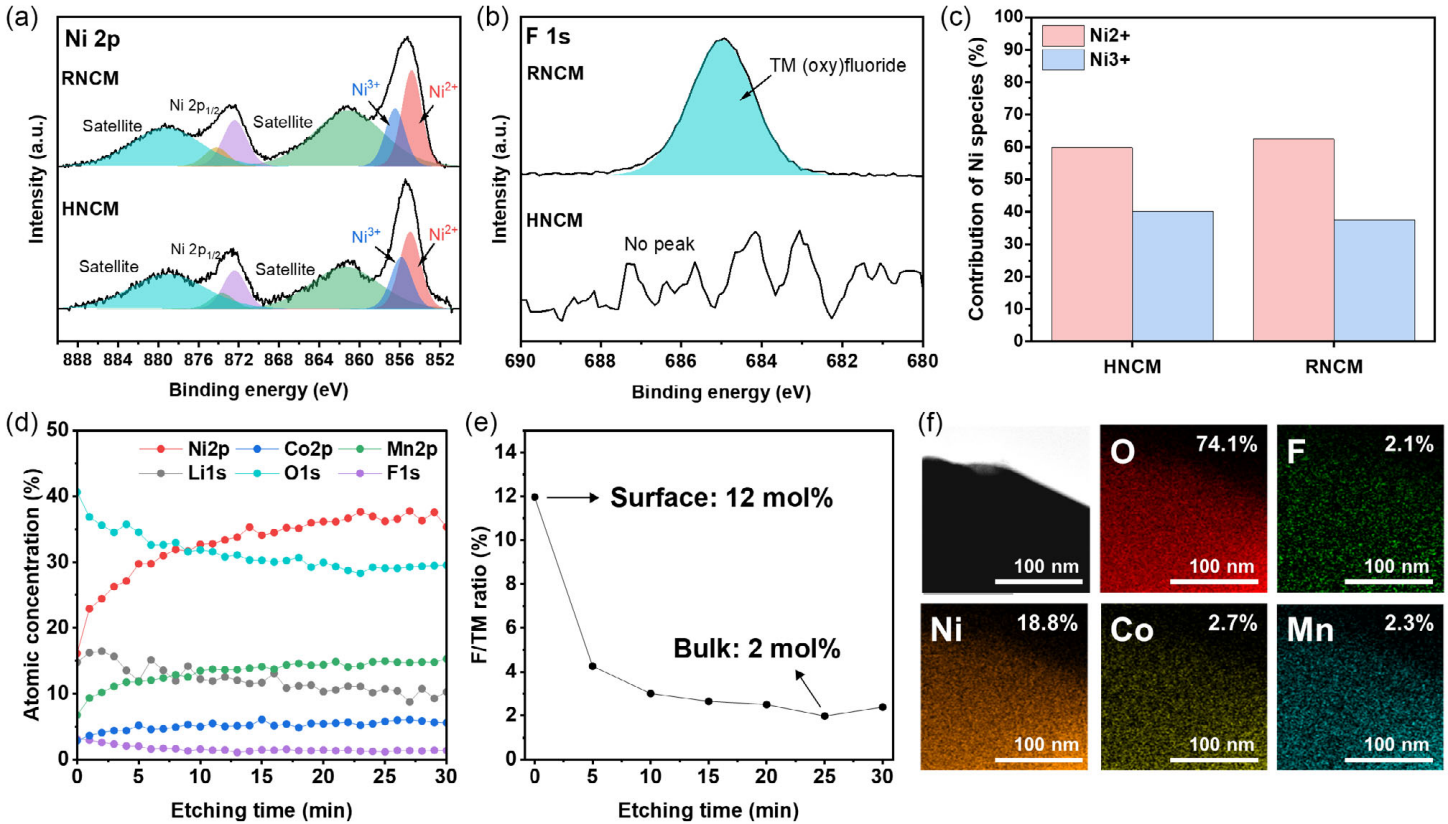
XPS spectra of HNCM and RNCM for a) Ni 2p, b) F 1s, and c) corresponding contribution of Ni^2+^ and Ni^3+^. d) XPS depth profile, e) F/TM ratio, and f) FE‐TEM EDS mapping images of RNCM.

XPS analysis was performed to confirm the differences in Ni oxidation states between HNCM and RNCM. In the Ni 2p spectra (**Figure** **6**a), peaks at 855 and 856 eV were assigned to Ni^2+^ and Ni^3+^, respectively.^[^ ^38^, ^39^ ^]^ Based on the deconvoluted peak areas, the relative compositions of Ni^2+^ and Ni^3+^ were calculated in Figure 6c. HNCM showed 59.82% Ni^2+^ and 40.17% Ni^3+^, while RNCM showed a higher proportion of Ni^2+^ at 62.47% and 37.52% of Ni^3+^, verifying the effect of F doping on Ni valence state. Furthermore, a peak attributed to transition metal (oxy)fluoride was detected near 685 eV in the RNCM sample (Figure 6b), suggesting the incorporation of F into the NCM lattice.^[^ ^40^ ^]^ However, no F 1s signal was detected in the HNCM sample (Figure 6b). The XPS results revealed an increased fraction of Ni^2+^ in RNCM, validating the earlier discussion that F doping induced a reduction of Ni oxidation states. These findings from XRD and XPS results imply that F40 used in RNCM contained fluorine that negatively affects the crystal structure during NCM synthesis, in agreement with the results presented in Figure 2 and 3.

To further clarify whether fluorine is only present on the surface or is located in the bulk, XPS depth profiling were carried out on RNCM (Figure 6d). The signal of F 1s persisted after sequential etching, confirming that fluorine species was not only adsorbed on the surface but also diffused into the bulk lattice. Quantitatively, the F/TM ratio was ≈12 mol% at the surface and ≈2 mol% in the bulk (Figure 6e), respectively. It is clear that the excess of threshold value (over 2 mol%) resulted in excessive cation mixing and structural degradation in Ni‐rich NCM,^[^ ^33^ ^]^ because recycled Li_2_CO_3_ introduces abnormal F doping (too high degree) during NCM preparation. In addition, FE‐TEM EDS mapping (Figure 6f) further confirmed a relatively uniform F distribution from the surface to the interior of RNCM particles, consistent with bulk incorporation. Due to the detection limitations of EDS, lithium is not directly observed and is instead represented as oxygen; consequently, the apparent concentration of F species (acquired from EDS) is lower than the value obtained from XPS analysis. Nevertheless, the combination between XPS depth profiling and mapping results well demonstrate incorporation of F species in RNCM (from surface to bulk). The excessive fluorine doping throughout the bulk level promotes severe cation mixing, aggravated lithium deficiency, and ultimately the formation of inhomogeneously agglomerated primary particles rather than well‐developed secondary structures.

### 2.3 Electrochemical Performances of HNCM and RNCM

**Figure 7:**
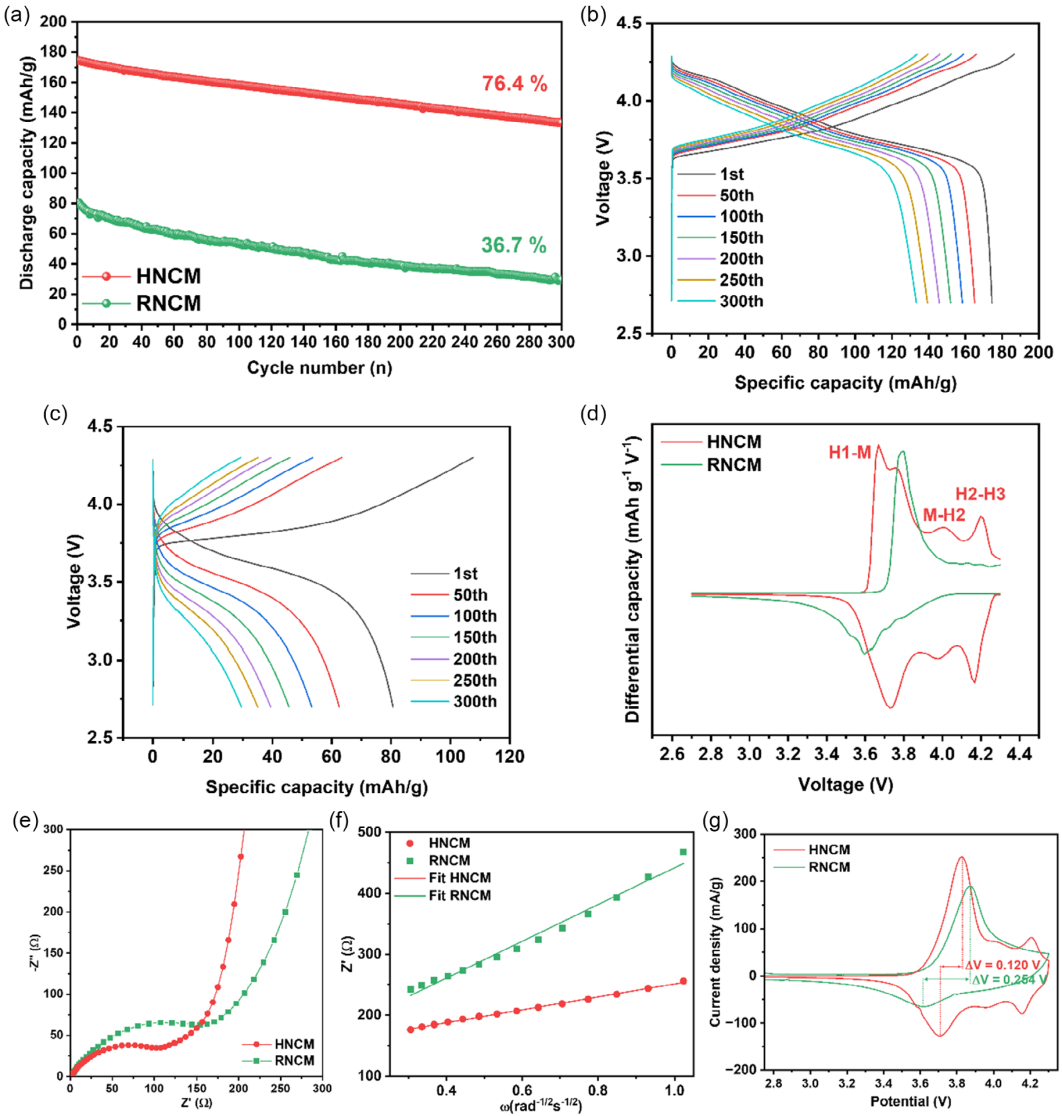
Electrochemical performances of HNCM and RNCM. a) Cycle performances at 0.5 C (1 C = 200 mAh g^−1^) for 300 cycles. b,c) Charge and discharge curves in the cut‐off voltage range of 2.7–4.3 V (vs. Li/Li^+^). d) dQ/dV curves for the 1st cycle. e) Nyquist plots before cycling. f) Relationship between real parts of the complex impedance and *ω* ^−1/2^. g) CV curves before cycling.

Half‐cells were assembled to evaluate the electrochemical performances of the synthesized HNCM and RNCM. As illustrated in **Figure** **7**a, RNCM has significantly poorer cycling stability compared to HNCM. HNCM delivered an initial discharge capacity of 174.5 mAh g^−1^ and retained 133.4 mAh g^−1^ after 300 cycles. By comparison, RNCM exhibited a much lower initial discharge capacity of 80.63 mAh g^−1^ and only 29.58 mAh g^−1^ after 300 cycles. This inferior performance is due to the high degree of cation mixing in RNCM. Li+ diffusion typically occurs via two mechanisms in NCM: oxygen dumbbell hopping (ODH)^[^ ^41^ ^]^ and tetrahedral site hopping (TSH). However, when cation mixing occurs, Ni ions occupy Li sites, introducing strong electrostatic repulsion that impedes Li^+^ migration through ODH pathways. In addition, the presence of Ni ions at Li antisites physically hinders TSH diffusion as well.^[^ ^42^ ^]^ Consequently, Li^+^ transport is suppressed during charge and discharge, resulting in lower capacity.

Figure 7a and b display the charge/discharge curves of HNCM and RNCM for 300 cycles. In comparison with HNCM, RNCM indicates a severe voltage drop during the cycle. The differential capacity curve for the first cycle in Figure 7c further highlights the structural changes. HNCM underwent a typical phase transition of high‐nickel NCM during charging—H1 (hexagonal) to M (monoclinic), M to H2 (hexagonal), H2 to H3 (hexagonal).^[^ ^43^, ^44^ ^]^ On the other hand, RNCM displays only a single peak during charging, deviating from the expected behavior for general F‐doped NCM.^[^ ^45^, ^46^ ^]^ This deviation suggests that structural and electrochemical degradation originate from the high fluorine content in RNCM.

Electrode kinetics were examined by performing electrochemical impedance spectroscopy (EIS) analysis before cycling. Figure 7e and f illustrate the Nyquist plots and corresponding Z′ versus *ω* ^−1/2^ plots for the HNCM and RNCM electrodes. The semicircle in the high‐frequency region of the Nyquist plot reflects the charge transfer resistance (*R*_ct_) at the electrode–electrolyte interface. As listed in Table 2, HNCM has a notably lower *R*_ct_ value (93.5 Ω) compared to RNCM (157 Ω). This enables HNCM to exhibit superior electrochemical performance, which is consistent with the results in Figure 7 (a–d). The higher R_ct_ of RNCM can be attributed to the increased cation mixing (Table 2), which likely hinders Li^+^ diffusion by blocking the Li^+^ migration pathways. In the low‐frequency region, the following equation was applied to extract the slope *σ* from the linear portion of the Z′ versus *ω* ^−1/2^ plot in Figure 7f.^[^ ^47^ ^]^

(5) $$\text{Z′}=R_{s}+R_{\text{ct}}+{\text{σω}}^{-1/2}$$

From the extracted *σ*, the lithium‐ion diffusion coefficient (*D*_Li_^+^) was calculated using the following equation.

(6) $$D_{{\text{Li}}^{+}}=\frac{R^{2}T^{2}}{{\text{2A}}^{2}n^{4}F^{4}C^{2}\sigma^{2}}$$

here, *R* denotes the gas constant, *T* is the absolute temperature, *A* is the electrode area, n is the number of electrons involved in the reaction, *F* is the Faraday constant, *C* is the Li^+^ concentration, and σ is the Warburg coefficient obtained from the slope. The calculated *D*_Li_^+^ values were 2.7 × 10^−12^ cm^2^ s^−1^ for HNCM and 3.29 × 10^−13^ cm^2^ s^−1^ for RNCM, demonstrating RNCM has a lower lithium‐ion diffusivity (**Table** **3**). CV was also employed to investigate the redox behavior of both samples before cycling. All measurements were conducted in the voltage range of 2.7–4.3 V (vs. Li/Li^+^) at a scan rate of 0.1 mV/s. As shown in Figure 7g, the polarization of HNCM was 0.120 V. By comparison, RNCM revealed a higher value of 0.254 V, which reflects more irreversibility and suggests limited long‐term stability. In addition, Figure S3 and Table S1, Supporting Information confirm that the trend between HNCM and RNCM is ever maintained after 300 cycles.

**Table 3:** Corresponding fitted *R*_ct_ and *D*_Li_^+^ values of HNCM and RNCM.

	HNCM	RNCM
*R*_ct_ [Ω]	93.5	157
*D*_Li_^+^ [cm^2^ s^−1^]	2.7 × 10^−12^	3.29 × 10^−13^

### 2.4 Verification of F Doping Effect on the Structure of RNCM

**Figure 8:**
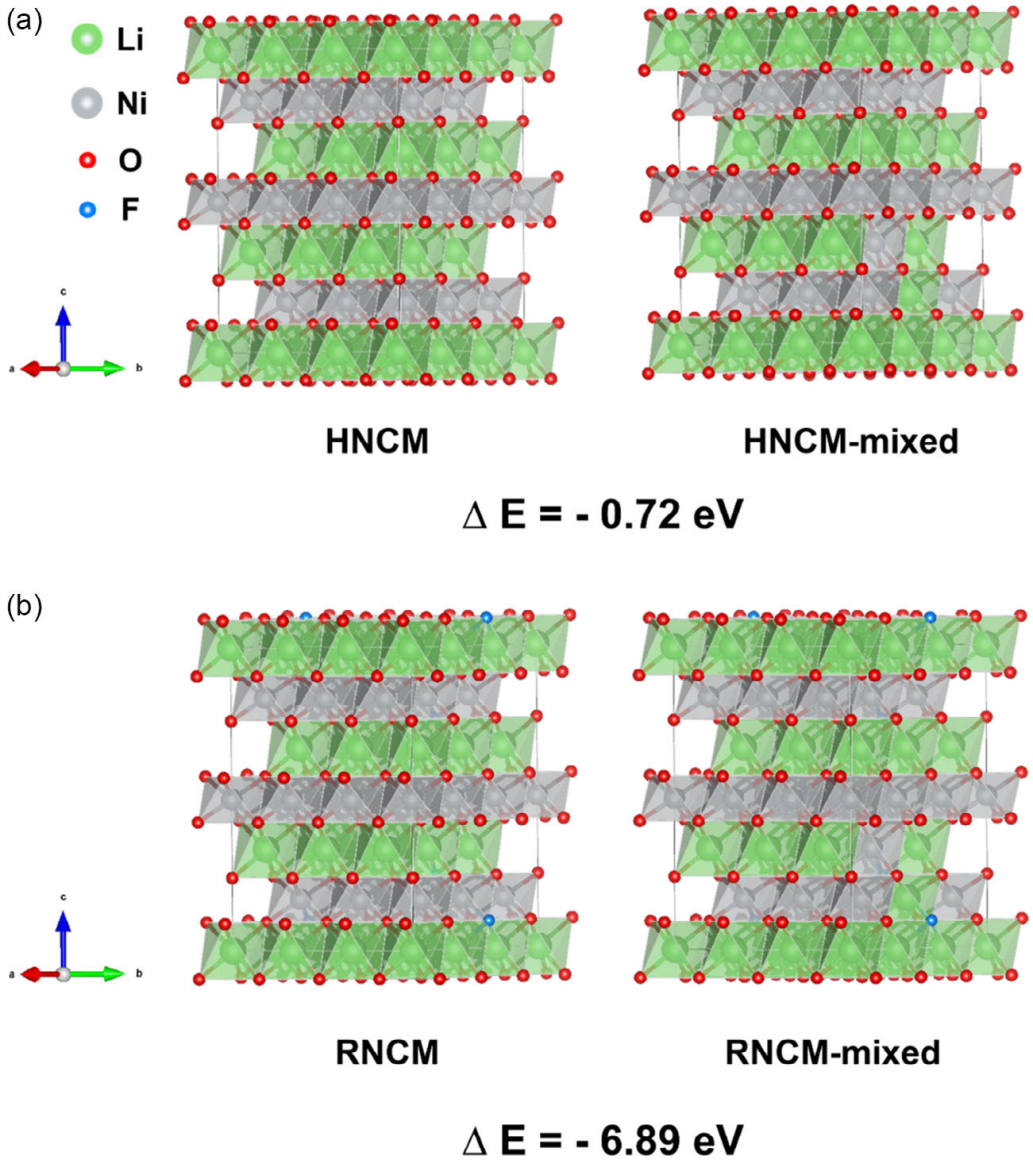
Structural diagram of formation energy of cation mixing for a) HNCM and b) RNCM.

The Ni/Li cation mixing energy barriers of HNCM and RNCM were evaluated using density functional theory (DFT) calculations. **Figure** **8**a and b depict the pristine and Ni/Li mixed structures of HNCM and RNCM, respectively. LiNiO_2_ (LNO) structure was adopted as a representation model for high‐nickel NCM due to its structural similarity and dominant Ni content. The formation energies of cation mixing were computed based on the following equations.^[^ ^48^ ^]^

(7) $$\Delta E_{\text{cation mixing}}=E_{{\left({\text{Li}}_{26}{\text{Ni}}_{1}\right)}_{\text{Li}}{\left({\text{Ni}}_{27}{\text{Li}}_{1}\right)}_{\text{Ni}}{\left(O_{54}\right)}_{O}}\;-E_{{\left({\text{Li}}_{27}\right)}_{\text{Li}}{\left({\text{Ni}}_{28}\right)}_{\text{Ni}}{\left(O_{54}\right)}_{O}}$$

(8) $$\Delta E_{\text{cation mixing}}=E_{{\left({\text{Li}}_{26}{\text{Ni}}_{1}\right)}_{\text{Li}}{\left({\text{Ni}}_{27}{\text{Li}}_{1}\right)}_{\text{Ni}}{\left(O_{50}F_{4}\right)}_{O}}\;-E_{{\left({\text{Li}}_{27}\right)}_{\text{Li}}{\left({\text{Ni}}_{28}\right)}_{\text{Ni}}{\left(O_{50}F_{4}\right)}_{O}}$$

The calculated energy barriers of cation mixing were 0.20 eV for HNCM and −1.15 eV for RNCM. These values suggest that cation mixing is thermodynamically favorable in RNCM because of its lower energy barrier than HNCM. This result supports the previous characterization findings, where excessive F doping in RNCM was correlated with increased structural instability and inferior material properties.

## 3 Conclusion

In this study, we demonstrated that spent commercial cathode materials can be sustainably recycled through a mechanochemical process without the need for high temperature or acidic/alkaline solutions. By directly employing a high‐energy ball mill and then reacting with DI water under mild heating, lithium (more specifically, Li‐salt) was successfully extracted through a simple and environmentally friendly procedure.

The extracted lithium salt was confirmed as Li_2_CO_3_, which is commonly used for cathode synthesis. Using this recycled lithium source, high‐nickel NCM was synthesized via a hydrothermally prepared precursor followed by calcination. To assess the quality of recycled Li_2_CO_3_, the electrochemical properties of HNCM (synthesized with reagent‐grade Li_2_CO_3_) and RNCM (synthesized with recycled Li_2_CO_3_) were compared. HNCM exhibited an initial discharge capacity of 174.5 mAh g^−1^ with a capacity retention of 76.4% after 300 cycles at 0.5 C. In contrast, RNCM delivered only 80.63 mAh g^−1^ initially and retained 36.7% of its capacity after 300 cycles.

The performance degradation in RNCM was ascribed to fluorine originating from the PVDF binder, which was not removed during ball milling. XRD, XPS, and quantitative analysis results confirmed the presence of fluorine in the recovered Li_2_CO_3_. Excessive fluorine doping during NCM synthesis caused severe cation mixing and structural distortion, which negatively affected electrochemical performance. This was further supported by DFT calculations, which demonstrated a significantly lower Ni/Li cation mixing energy barrier for RNCM compared to HNCM.

Altogether, this study extends previous work–limited to reagent‐grade cathode materials and Al foil–by showing that even commercial NCM cathodes can be recycled by a simple, environmentally friendly mechanochemical process. Future studies will be needed to remove fluorine effectively to enhance the practical viability of this sustainable LIB recycling strategy.

## Supporting Information

The authors have cited additional references within the Supporting Information.

## Conflict of Interest

The authors declare no conflict of interest.

## Supporting information

Supplementary Material

## Data Availability

The data that support the findings of this study are available from the corresponding author upon reasonable request.
