# Supplementary material for: Lithium Recycling from Spent NCM523 Cathode and Resynthesis of High‐Nickel Lithium‐Ion Battery Using Recovered Material
Source: ChemSusChem. 2025 Oct 28;18(24):e202501510. doi: 10.1002/cssc.202501510 (PMC12703445; doi:10.1002/cssc.202501510)
Supplement: Supplementary file 1 — Supplementary Material [file CSSC-18-e202501510-s001.pdf]

Supporting Information

Lithium Recycling from Spent NCM523 Cathode and Resynthesis of High-Nickel Li-ion Battery Using Recovered Material

Jinwon Seo+ [a], Hyerin Jeon+ [a], Hui-il Nam[b], Jinhee Lee*[a], Yong-Wook Choi*[b], and Jinsub Choi*[a]

[a]*Department of Chemistry and Chemical Engineering, Inha University, Michuhol-gu, 22212 Incheon, Republic of Korea*

[b]*Energy System Group, Korea Institute of Industrial Technology (KITECH), Busan, 46938, Republic of Korea*

**Corresponding authors: yongwook@kitech.re.kr, ezi333@inha.edu and jinsub@inha.ac.kr*

Correspondence and request for materials should be addressed to Yong-Wook Choi (email: yongwook@kitech.re.kr) and Jinsub Choi (email: jinsub@inha.ac.kr).

Experimental details

1 Collecting NCM cathode materials from degraded 18650 LIB

The NCM523 cathodes were separated from the commercially purchased 18650 LIB (lithium ion battery). All 18650 cells were degraded after 2000 charge/discharge cycles. To ensure complete discharge to 0% state of charge (SoC), cells were immersed in a 10 wt% NaCl (99.5%, JUNSEI) solution for 24 h. Then the 18650 cells were disassembled to collect cathode materials. All collected cathode sheets were dried under a fume hood overnight.

2 Extracting lithium from collected NCM

The collected NCM523 cathode materials were manually cut into smaller pieces. A total of 2 g of cut NCM523 was placed into a 50 ml stainless steel jar (Retsch) along with stainless steel balls at varying ball-to-powder ratios (BPRs). Then the high-energy ball milling was conducted for 6 h, 500 rpm using a PM100 planetary ball mill (Retsch). After ball milling, all powder was transferred to a water-jacketed reactor containing 250 ml of deionized (DI) water. The mixture was stirred at 95 °C, 300 rpm for 2 h. The resulting material was dried overnight at 70 °C. After drying, it was dispersed in 100 ml of DI water and stirred for 10 min. The resulting suspension was then filtered using an aspirator and a PTFE membrane to separate the residue from the filtrate. The collected filtrate was subsequently dried overnight at 70 °C. As a result, lithium was extracted and labeled as Fxx, with “xx” indicating the applied BPR (e.g. F40 for a BPR of 40:1). Following the filtration, the residue was confirmed to contain Al, Ni, Co, and Mn.

3 Synthesis of high-nickel cathode

Hydrothermal method was used to prepare high-nickel NCM (NCM811) precursors. Nickel (II) sulfate hexahydrate ($NiSO_4 \cdot 6H_2O$, >98%, Sigma-Aldrich), cobalt (II) sulfate heptahydrate ($CoSO_4 \cdot 7H_2O$, ≥99%, Sigma-Aldrich), manganese (II) sulfate monohydrate ($MnSO_4 \cdot H_2O$, ≥99%, Sigma-Aldrich) were dissolved in a mixed solution of DI water and ethylene glycol (99.5%, SAMCHUN) under stirring at 300 rpm for 40 min. After dissolution, a stoichiometric amount of ammonium bicarbonate (NH_4HCO_3, 99%, Acros Organics) was added to a solution

under continuous stirring until complete precipitation. Afterwards, the mixture was transferred to a 100 ml Teflon-lined autoclave and hydrothermally reacted at 200 ℃ for 24 h in a muffle furnace with a heating rate of 5 ℃ min^{-1}. Once the solution was cooled to room temperature, the precipitate was washed several times with DI water and absolute ethanol, followed by drying at 80 ℃ for 12 h. To synthesize NCM811 ($LiNi_{0.8}Co_{0.1}Mn_{0.1}O_2$), dried precursor was mixed with reagent grade Li_2CO_3 (HNCM) and F40 (RNCM) respectively at a molar ratio of TM : Li = 1 : 1.06. The mixture was pre-calcined at 500 ℃ for 6 h and then calcined at 780 ℃ for 15 h under an oxygen atmosphere in a tube furnace.

4 Materials characterization

Field-emission scanning electron microscopy (FE-SEM; S-4300, Hitachi) analyses were performed to examine the morphologies of extracted lithium (Fxx), HNCM and RNCM. Their crystal structures were identified by Field-emission transmission electron microscopy (FE-TEM; JEM-2100F, JEOL) and X-ray diffraction (XRD; Smartlab SE, Rigaku). Chemical states of F40, HNCM and RNCM were analyzed by X-ray photoelectron spectroscopy (XPS; Nexsa-G2, ThermoFisher Scientific). Additionally, the doping state of RNCM was investigated with ion etching. For the quantitative investigation of extracted lithium, atomic absorption spectrometry (AAS; AAnalyst 400, PerkinElmer), inductively coupled plasma optical emission spectrometry (ICP-OES; Optima 7300DV, PerkinElmer), and ion chromatography (IC; ICS-3000, Dionex) were employed.

5 Coin cell fabrication and electrochemical measurements

The prepared HNCM and RNCM active materials with a conductive agent (Super P) and binder (polyvinylidene fluoride, PVDF) were mixed in a weight ratio of 8:1:1 and dispersed in N-methyl-2-pyrrolidone (NMP). The mixed slurry was cast onto an aluminum foil and dried under vacuum conditions at 80 ℃ for 12 h and 14 mm-diameter disks were punched from dried electrodes. The active material loading of the prepared electrodes was 1.5-1.8 mg. Electrochemical measurements were carried out in a half-cell configuration using CR2032-type coin cells in an Ar-filled glovebox. A 1 M $LiPF_6$ solution in EC/DEC (1:1 v/v) was used as the electrolyte, Celgard 2400 as the separator, and lithium metal as the counter electrode. Charge/discharge cycling was performed in a voltage range of 2.7-4.3 V (vs. Li/Li^+) using a

battery cycler system (CT-4008T, Sinopro). Cyclic voltammetry (CV) was conducted in the voltage range of 2.7-4.3 V (vs. Li/Li^+) at a scan rate of 0.1 mV s^{-1}, and electrochemical impedance spectroscopy (EIS) was carried out from 10^5 to 10^{-2} Hz using a potentio/galvanostat (Autolab PGSTAT128N, Metrohm).

6 DFT calculations

Rietveld refinement was performed using the REFLEX module in Materials Studio program (Accelrys Inc.). The reference crystal structure was based on the $LiNiO_2$ with space group $R\bar{3}m$. Density functional theory (DFT) calculations of cation mixing formation energies of HNCM and RNCM were performed using the CASTEP module in Materials Studio. DFT calculations were conducted using the PBE-GGA exchange-correlation functional with Hubbard U correction (GGA+U). A $Li_{27}Ni_{27}O_{53}$ and $Li_{27}Ni_{27}O_{50}F_4$ supercell were constructed to calculate the cation mixing energy of HNCM and RNCM. Hubbard U parameter for Ni was set to 6.5 eV.[1] The energy cutoff was 340 eV and ultrasoft pseudopotential was used. The convergence criteria were set as follows: energy = 1.0 x 10^{-5} eV, max force = 0.03 eV/Å, max stress = 0.05 GPa, max displacement = 0.001 Å. The k-point grid was set to 1 x 1 x 1 and the self-consistent field (SCF) tolerance was 1 x 10^{-6} eV/atom.

Additional experimental data

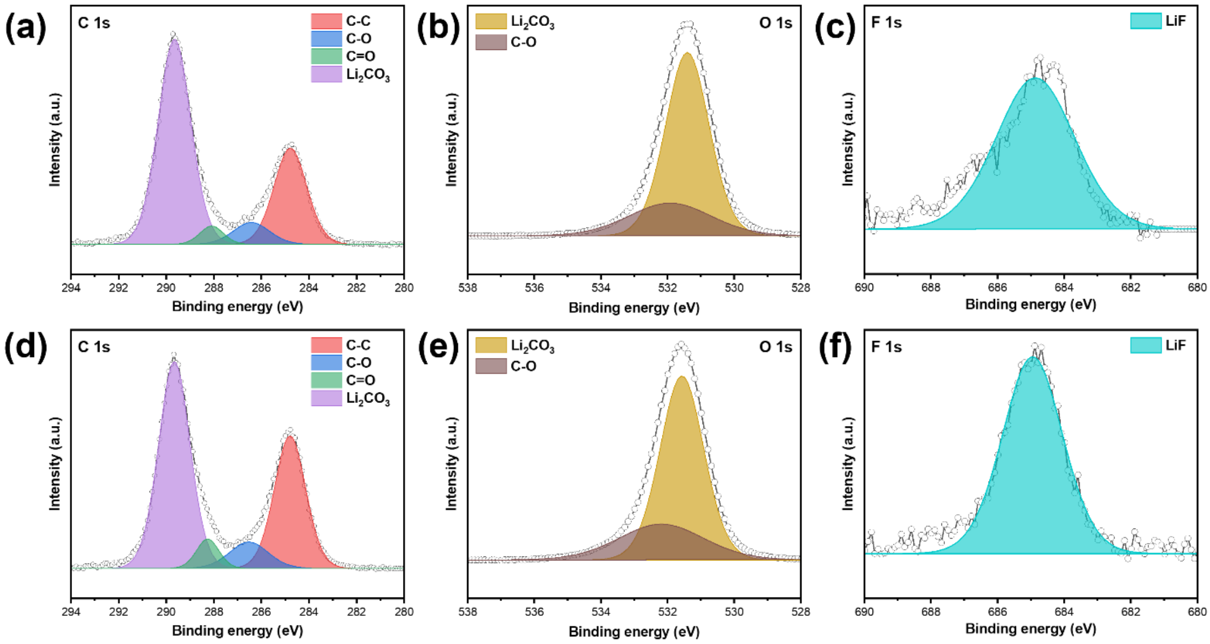

Figure S1. XPS spectra of (a-c) F20 sample and (d-f) F30 sample. (a,d) C 1S, (b,e) O 1S, and (c,f) F 1s of both samples.

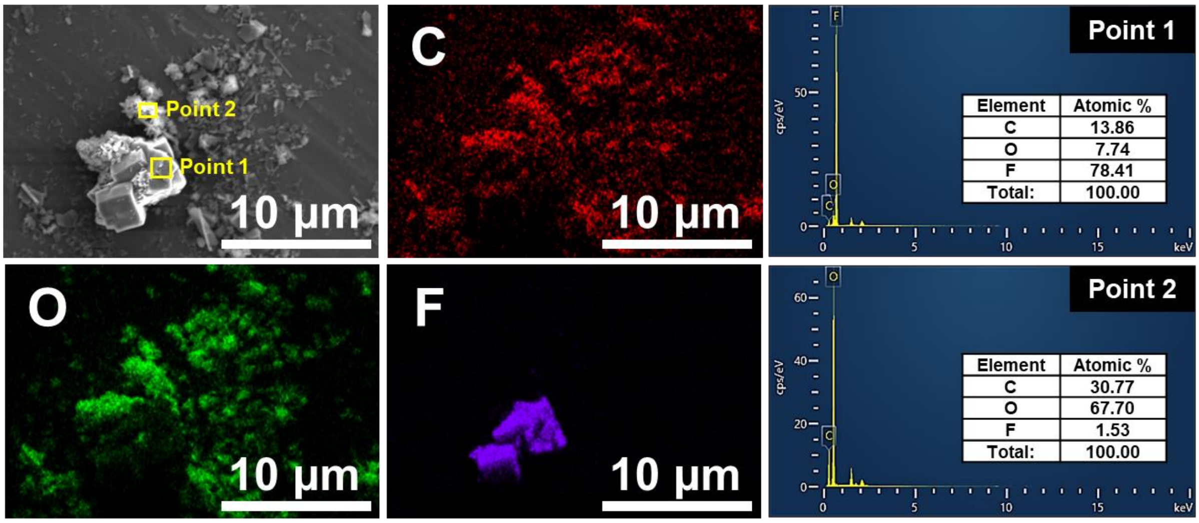

Figure S2. SEM EDS mapping and point EDS analyses of F40.

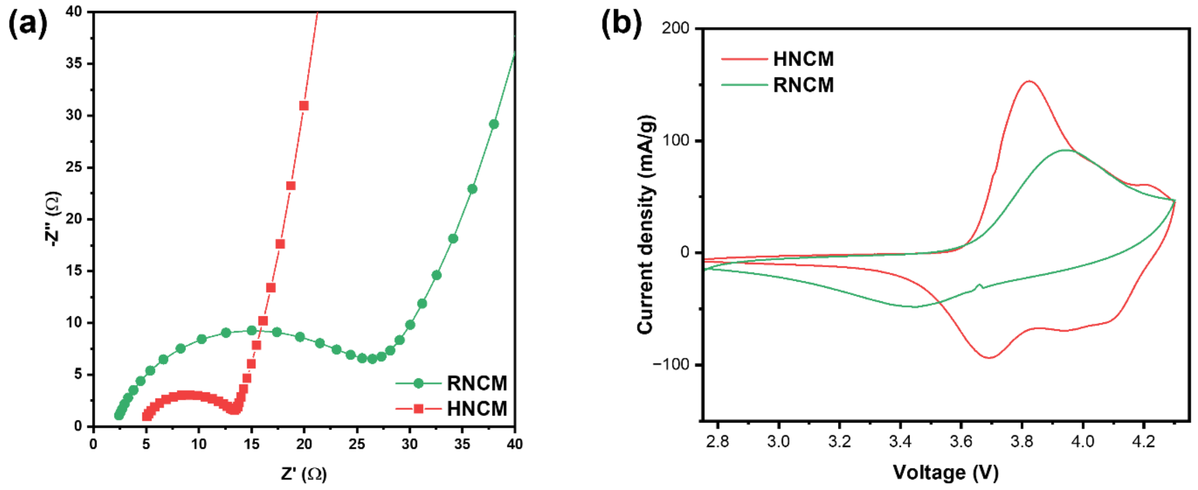

Figure S3. (a) Nyquist plots and (b) CV curves after 300 cycles of HNCM (red line) and RNCM (green) line.

Table S1. The values of solution resistance (R_s) charge-transfer resistance (R_{ct}), and polarization (ΔV) on both HNCM and RNCM.

	HNCM	RNCM
Rs (Ω)	4.461	1.971
Rct (Ω)	14.115	29.031
ΔV (V)	0.131	0.491

References

[1] H. Chen, J. A. Dawson, J. H. Harding, *J. Mater. Chem. A* **2014**, *2*, 7988.
